# Supplementary figures and images for: Genetic barcoding uncovers the clonal makeup of solid and liquid biopsies and their ability to capture intra-tumoral heterogeneity
Source: Mol Syst Biol. 2026 Feb 11;22(5):659–84. doi: 10.1038/s44320-026-00194-w (PMC13144504; doi:10.1038/s44320-026-00194-w)

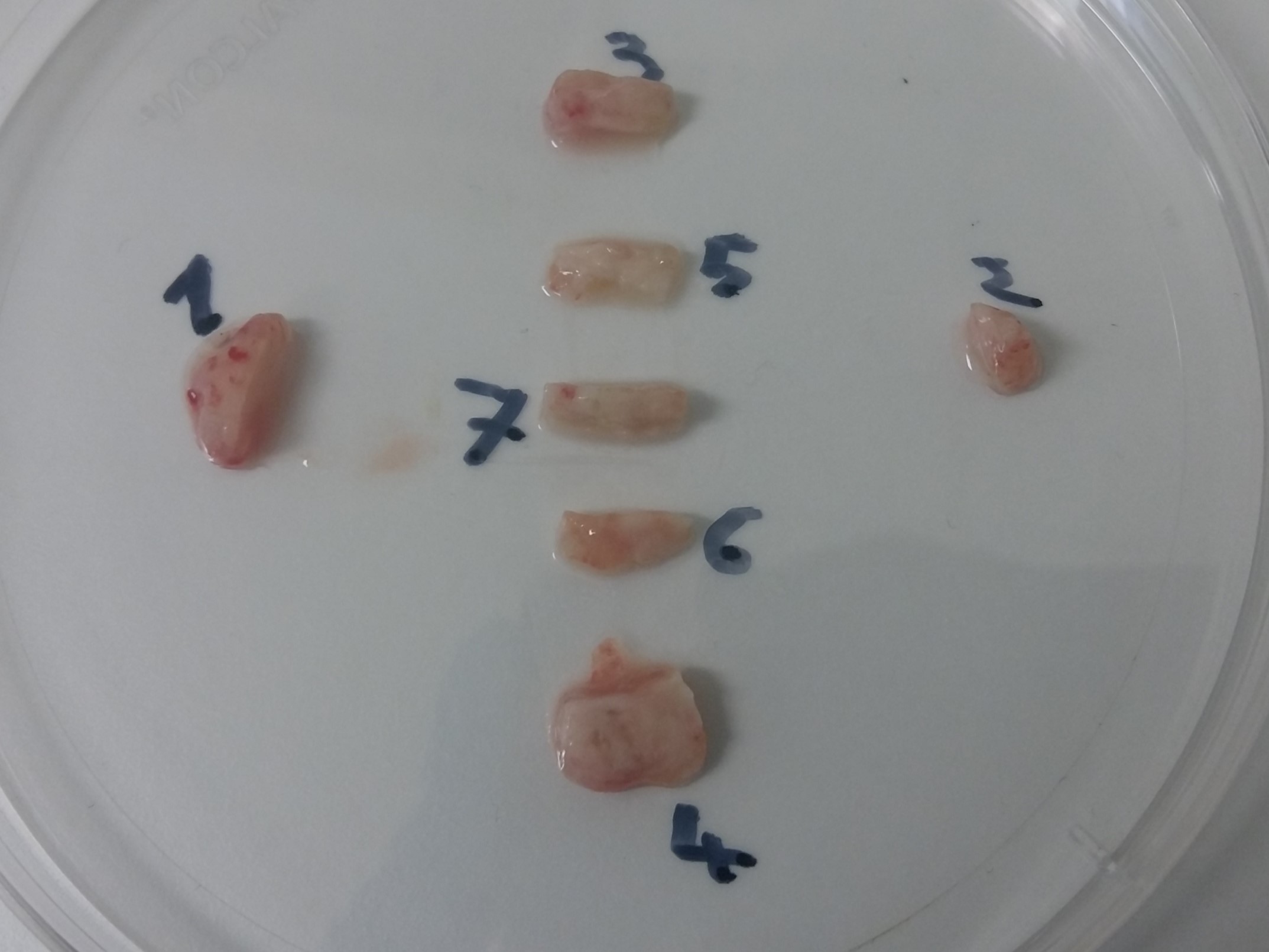

Supplement: Supplementary file 4 — Source data Fig. 1 [file 44320_2026_194_MOESM4_ESM.zip › Figure1/1C/152917.jpg]

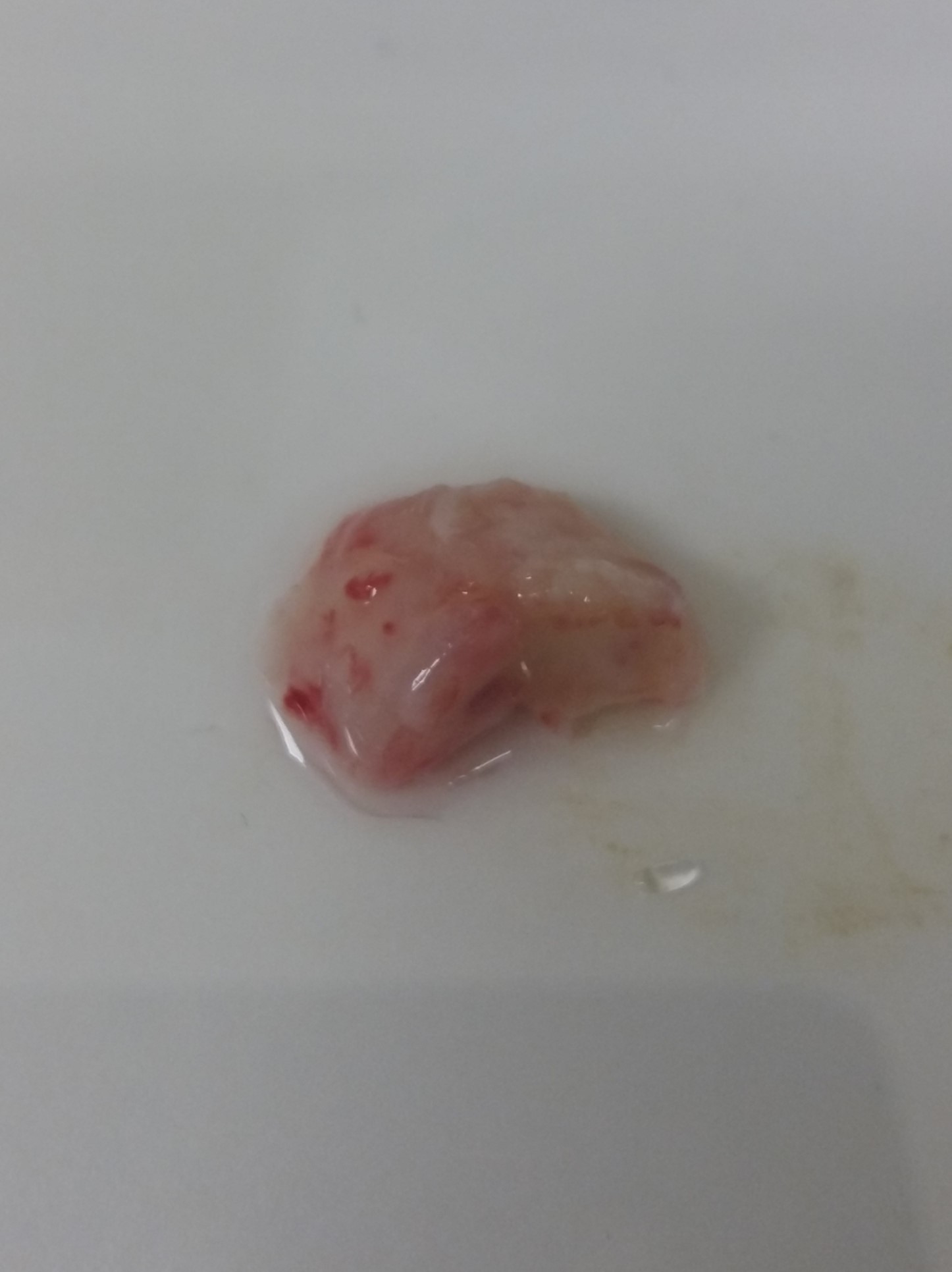

Supplement: Supplementary file 4 — Source data Fig. 1 [file 44320_2026_194_MOESM4_ESM.zip › Figure1/1C/152357.jpg]
