# Supplementary material for: Genetic barcoding uncovers the clonal makeup of solid and liquid biopsies and their ability to capture intra-tumoral heterogeneity
Source: Mol Syst Biol. 2026 Feb 11;22(5):659–84. doi: 10.1038/s44320-026-00194-w (PMC13144504; doi:10.1038/s44320-026-00194-w)
Supplement: Supplementary file 8 — Expanded View Figures [file 44320_2026_194_MOESM8_ESM.pdf]

Expanded View Figures

Figure EV1. Analysis of the barcode repertoire in primary tumors.

(**A**) Total number of barcodes detected in each tumor. Each dot represents a tumor sample. The average number of barcodes per tumor is indicated on top of the bars. (**B**) Shannon diversity index of barcoded tumors. Significance from one-way ANOVA followed by Tukey multiple comparisons test, *P* values: MDA-MB-231/PDX-1432C = 0.0070, MDA-MB-231/CRCM412 = 0.0320, MDA-MB-231/CRCM434 = 0.0003, MDA-MB-468/CRCM434 = 0.0443, PDX-1432C/PDX-4295 = 0.0254, PDX-4295/CRCM434 = 0.0022. **P* value < 0.05, ***P* value < 0.01, ****P* value < 0.001, *****P* value < 0.0001. (**A**, **B**) Each dot corresponds to a tumor. Error bars represent standard deviation (SD) of the mean. MDA-MB-231 were shortened to MDA-231, and MDA-MB-468 were shortened to MDA-468. (**C–F**) Example of primary tumor cutting and barcode composition for one mouse per model, from top to bottom, MDA-MB-231, MDA-MB-468, CRCM434, PDX-4295, PDX-1432C, CRCM412. (**C**) Photos of tumors collected in PBS and cut into pieces of similar size. (**D**) Representation of the clonal composition of each piece. (**E**) Heatmap representing the clonal composition of each peripheral piece (normal font), center pieces (red) and full tumor (bold). Each column represents a barcode, and its frequency is represented by the color scale. (**F**) Relationship between the percentage of barcodes detected in each tumor piece and the weight of the tumor piece. Each dot corresponds to a tumor piece, from the periphery (purple dots) or the center (orange dots). (**A**, **B**) MDA-MB-231, three independent experiments, *n* = 5, 4, 4, MDA-MB-468 one experiment *n* = 6, PDX-1432C two independent experiments, *n* = 6, 4, PDX-4295 one experiment *n* = 6, CRCM434 four independent experiments, *n* = 7, 6, 4, 5, CRCM412 four independent experiments, *n* = 4, 2, 5, 5.

 © The Author(s)

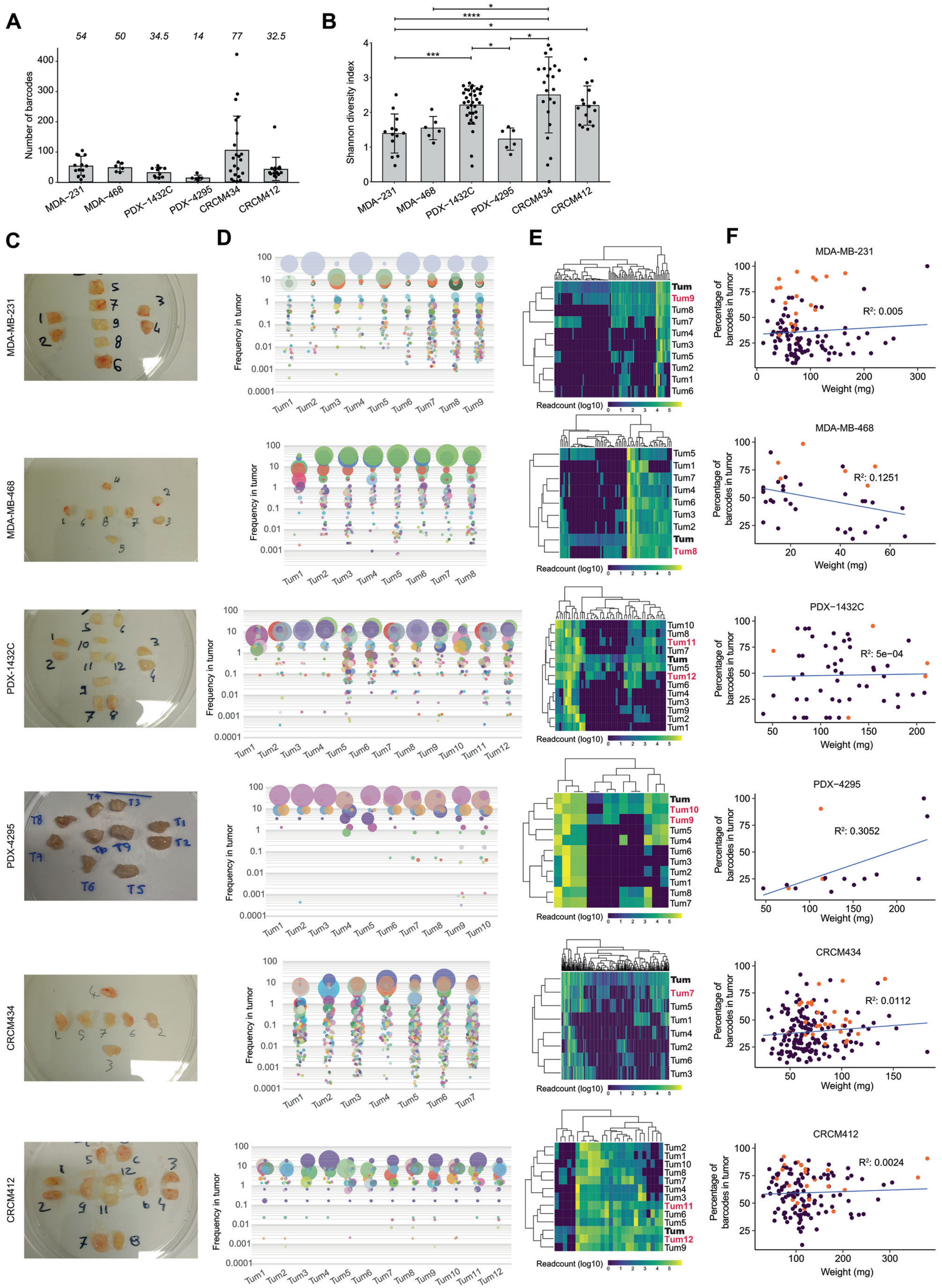
A
54 50 34.5 14 77 32.5
Number of barcodes
MDA-231 MDA-468 PDX-1432C PDX-4295 CRCM434 CRCM412
B
Shannon diversity index
***
*
*
*
****
*
C
MDA-MB-231
MDA-MB-468
PDX-1432C
PDX-4295
CRCM434
CRCM412
D
Frequency in tumor
E
Readcount (log10)
F
MDA-MB-231
Percentage of barcodes in tumor
Weight (mg)
R²: 0.005
MDA-MB-468
R²: 0.1251
PDX-1432C
R²: 5e-04
PDX-4295
R²: 0.3052
CRCM434
R²: 0.0112
CRCM412
R²: 0.0024

© The Author(s)

A

ID	Sample	Subtype	Hormone status			Comments on necrosis
			ER	PR	HER2	
1432C	Patient	TNBC	Neg	Neg	Neg	Extensive necrosis
1432C	PDX	TNBC	Neg	Neg	Neg	Extensively necrotic
CRCM412	Patient	TNBC	Neg	Neg	Neg	*Not available*
CRCM412	PDX	TNBC	Neg	Neg	Neg	Patchy necrosis
CRCM434	Patient	TNBC	Neg	Neg	Neg	*Not available*
CRCM434	PDX	TNBC	Neg	Neg	Neg	No comment on necrosis
4295	Patient	TNBC	Neg	Neg	Neg	*Not available*
4295	PDX	TNBC	Neg	Neg	Neg	No comment on necrosis

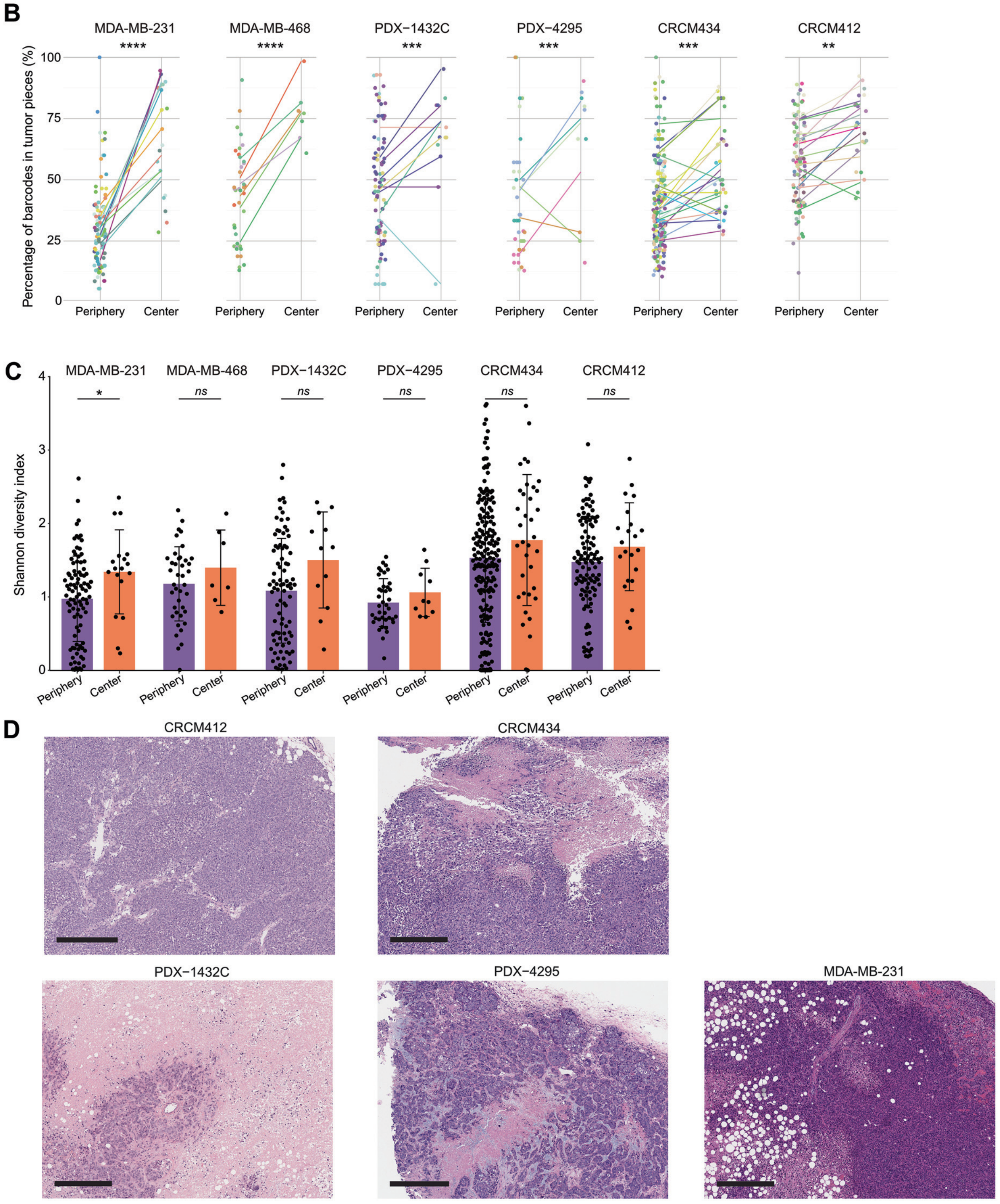

 © The Author(s)

Figure EV2. Characterisation of primary tumors.

(**A**) Breast cancer subtype and hormone receptors status from patient samples and matching PDXs. Estrogen receptor (ER), Progesterone receptor (PR) Human epidermal growth factor receptor 2 (HER2). Comments on necrosis were shown when available on patient's pathology reports and PDXs. Non-barcoded primary tumors from previous PDX passages were used for analysis. (**B**) Mixed-effect model analysis of barcode distribution across tumor locations and models. Each color represents a specific mouse, each dot corresponds to a tumor piece, and the line represents the linear interpolation between average for each tumor. Pairwise Wald *t* tests (linear mixed-effects model) between "Center" and "Periphery" locations within each tumor model, *P* values: MDA-MB-231 = 2.7e-16, MDA-MD-468 = 7.7e-07, PDX-1432C = 0.0007, PDX-4295 = 0.0005, CRCM434 = 0.0002, CRCM412 = 0.0057. **P* value < 0.05, ***P* value < 0.01, ****P* value < 0.001, *****P* value < 0.0001. (**C**) Shannon diversity index of tumor pieces from periphery (purple) or center (orange). Student's unpaired *t* test, *P* values: MDA-MB-231 = 0.0203, MDA-MD-468 = 0.3239, PDX-1432C = 0.0576, PDX-4295 = 0.2478, CRCM434 = 0.0868, CRCM412 = 0.1511. Error bars represent standard deviation (SD) of the mean. (**D**) H&E staining of primary tumors, scale bar 400 µm. (**B**, **C**) MDA-MB-231, three independent experiments, *n* = 5, 4, 4, MDA-MB-468 one experiment *n* = 6, PDX-1432C two independent experiments, *n* = 6, 4, PDX-4295 one experiment *n* = 6, CRCM434 four independent experiments, *n* = 7, 6, 4, 5, CRCM412 four independent experiments, *n* = 4, 2, 5, 5.

© The Author(s)

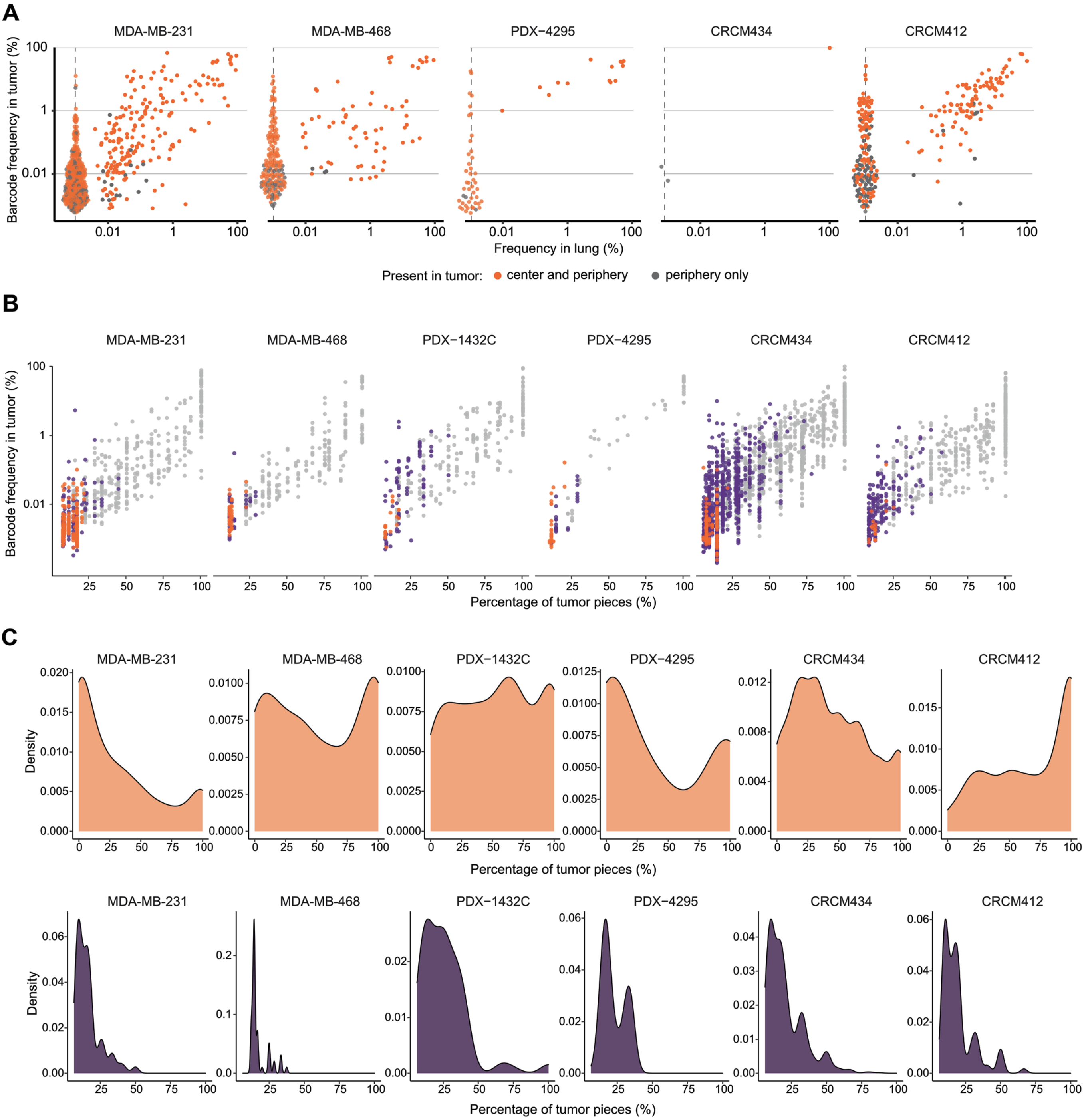

Figure EV3. Barcode distribution in primary tumor pieces and lungs.

(**A**) Clonal relationship between barcodes detected in primary tumors and lungs. The color indicates the localization of the barcodes. If the barcodes were found in the center of the primary tumor (not exclusively), the dots are orange. If the barcodes were localized exclusively in peripheral pieces, the dots are gray. Each dot represents a barcode. Dots on dashed lines represent barcode uniquely found in tumor and not detected in lung. MDA-MB-231, three independent experiments, $n = 1, 4, 4$, MDA-MB-468 one experiment, $n = 5$, PDX-4295 two experiments, $n = 1, 2$, CRCM434 one experiment, $n = 1$, CRCM412 three independent experiments, $n = 2, 1, 4$. (**B**) Relationship between the frequency of individual barcodes in primary tumors and the number of pieces (in percentage) containing these barcodes. Each dot represents a barcode uniquely found in the tumor center (orange), periphery (purple) or found in center and periphery (gray). (**C**) Density plot showing the distribution of the number of pieces (in percentage) from the periphery containing barcodes present in the center (top), or barcodes uniquely found in peripheral pieces (bottom). (**B**, **C**) MDA-MB-231, three independent experiments, $n = 5, 4, 4$, MDA-MB-468 one experiment $n = 6$, PDX-1432C two independent experiments, $n = 6, 4$, PDX-4295 one experiment $n = 6$, CRCM434 four independent experiments, $n = 7, 6, 4, 5$, CRCM412 four independent experiments, $n = 4, 2, 5, 5$.

 © The Author(s)

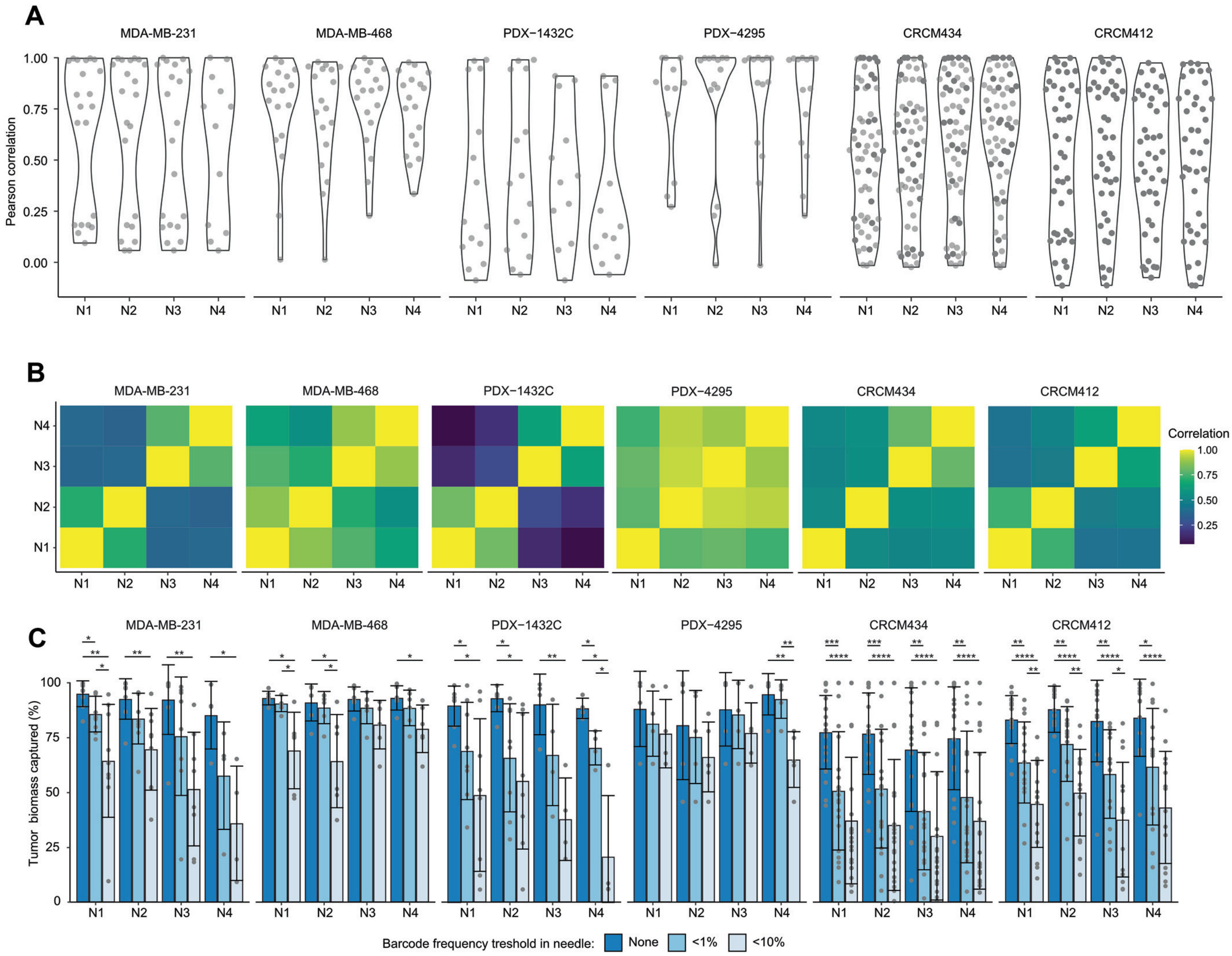

Figure EV4. Barcode correlation in solid biopsies.

(**A**) Correlation of clonal frequencies between needle samples and matching primary tumors. (**B**) Pearson correlation mean comparison between different needle samples from matching tumors represented as heatmap. (**C**) Percentage of primary tumor biomass captured in needle samples without threshold (left blue bar), for barcode frequency above 1% (middle bar), or above 10% (right bar). Student's unpaired *t* test, **P* value < 0.05, ***P* value < 0.01, ****P* value < 0.001, *****P* value < 0.0001. Error bars represent standard deviation (SD) of the mean. Each dot corresponds to a needle biopsy. MDA-MB-231 three independent experiments, *n* = 1, 4, 4, MDA-MB-468 one experiment, *n* = 6, PDX-1432C two independent experiments, *n* = 4, 4, PDX-4295 one experiment, *n* = 5, CRCM434 four independent experiments, *n* = 7, 6, 4, 5, CRCM412 four independent experiments, *n* = 4, 2, 5, 5. *P* values: MDA-MB-231 N1 No threshold-1% = 0.0145, N1 No threshold-10% = 0.0072, N1 1%-10% = 0.0407, N2 No threshold-10% = 0.0064, N3 No threshold-10% = 0.0027, N4 No threshold-10% = 0.0235; MDA-MB-468 N1 No threshold-10% = 0.0195, N1 1%-10% = 0.0290, N2 No threshold-10% = 0.0260, N2 1%-10% = 0.0365, N4 No threshold-10% = 0.0237; PDX-1432C N1 No threshold-1% = 0.0383, N1 No threshold-10% = 0.0129, N2 No threshold-1% = 0.0167, N2 No threshold-10% = 0.0107, N3 No threshold-10% = 0.0051, N4 No threshold-1% = 0.0110, N4 No threshold-10% = 0.0153, N4 1%-10% = 0.0332; PDX-4295 N4 No threshold-10% = 0.0035, N4 1%-10% = 0.0051; CRCM434 N1 No threshold-1% = 0.0005, N1 No threshold-10% = 4.2e-06, N2 No threshold-1% = 0.0009, N2 No threshold-10% = 2.9e-06, N3 No threshold-1% = 0.0020, N3 No threshold-10% = 7.1e-05, N4 No threshold-1% = 0.0021, N4 No threshold-10% = 5.5e-05; CRCM412 N1 No threshold-1% = 0.0013, N1 No threshold-10% = 5.8e-07, N1 1%-10% = 0.0092, N2 No threshold-1% = 0.0040, N2 No threshold-10% = 6.4e-07, N2 1%-10% = 0.0020, N3 No threshold-1% = 0.0029, N3 No threshold-10% = 2.4e-05, N3 1%-10% = 0.0268, N4 No threshold-1% = 0.0154, N4 No threshold-10% = 5.4e-05.

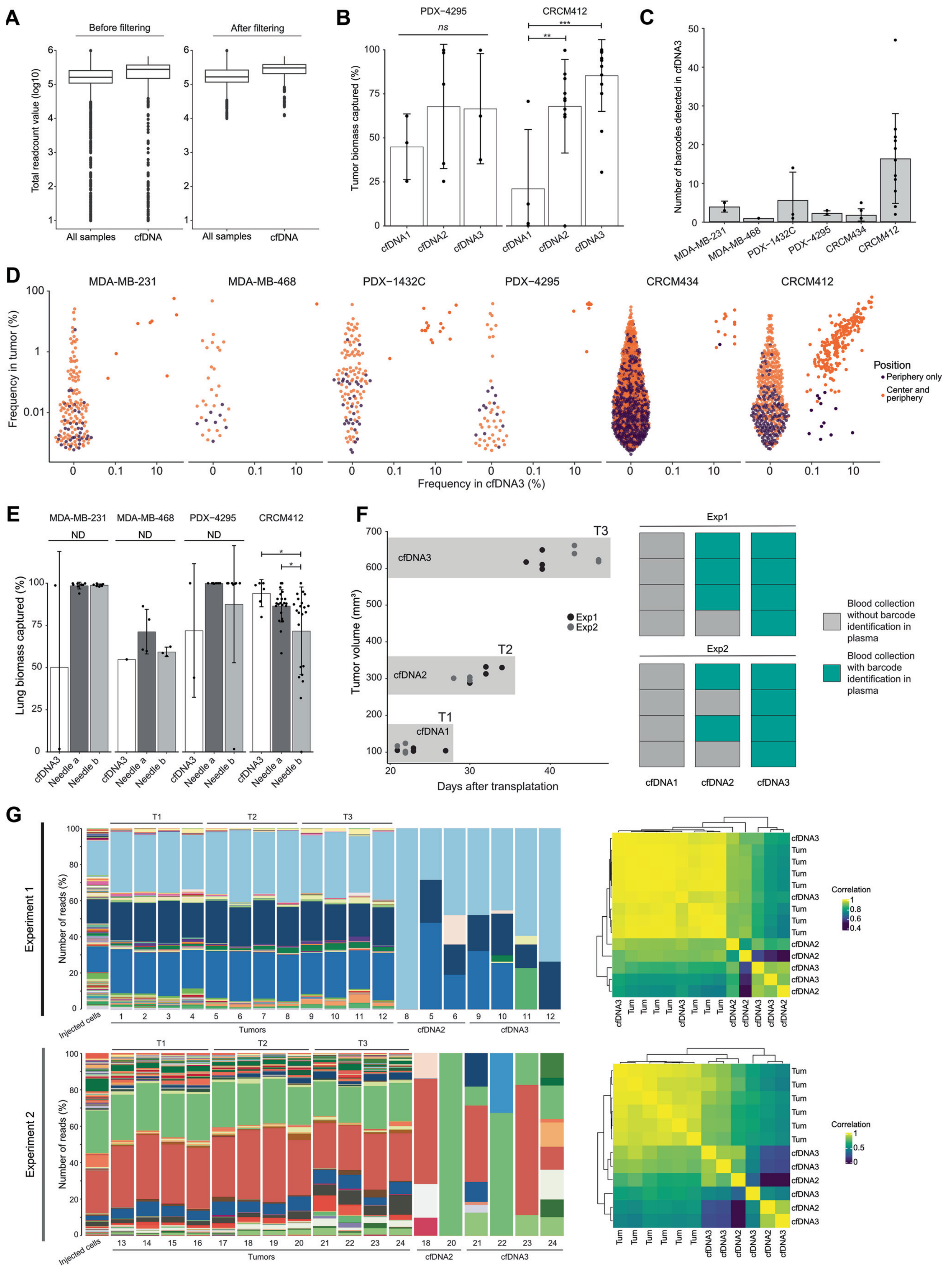
A
Before filtering
After filtering
Total readcount value (log10)
All samples
cfDNA
B
PDX−4295
CRCM412
ns
Tumor biomass captured (%)
cfDNA1
cfDNA2
cfDNA3
C
Number of barcodes detected in cfDNA3
MDA-MB-231
MDA-MB-468
PDX−1432C
PDX−4295
CRCM434
CRCM412
D
Frequency in tumor (%)
Frequency in cfDNA3 (%)
Position
Periphery only
Center and periphery
E
ND
Lung biomass captured (%)
Needle a
Needle b
F
Tumor volume (mm³)
Days after transplatation
T1
T2
T3
Exp1
Exp2
Blood collection without barcode identification in plasma
Blood collection with barcode identification in plasma
G
Experiment 1
Experiment 2
Number of reads (%)
Injected cells
Tumors
Tum
Correlation

Figure EV5. Barcode analysis in cfDNA.

(**A**) Barcoded sample coverage, highlighting specific read-count values for cfDNA samples versus all samples. Total read count per sample, before (left) and after filtering (right). Boxplots show the median (center line), interquartile range (box; 25th–75th percentiles), and whiskers indicate minima and maxima extending to x1.5 the interquartile range, outliers shown as individual points. (**B**) Percentage of tumor biomass captured at different timepoints. One-way Anova followed by Tukey multiple comparisons test, *P* values CRCM412 cfDNA1-cfDNA2 = 0.0096, cfDNA1-cfDNA3 = 0.0003. Error bars represent standard deviation (SD) of the mean, PDX-4295 two independent experiments, *n* = 1, 4, CRCM412 four independent experiments, 4, 2, 5, 4. Each dot corresponds to a cfDNA sample. (**C**) Number of barcodes detected in cfDNA3 in different models. Each dot corresponds to a cfDNA sample. Error bars represent standard deviation (SD) of the mean. (**D**) Relationship between the barcode in primary tumor and cfDNA3. Each barcode is represented by a dot, and its color represents its location in the primary tumor. Barcodes exclusively detected in peripheral pieces are represented in purple and barcodes detected in the center are represented in orange. (**C**, **D**) MDA-MB-231 one experiment, *n* = 2, MDA-MB-468 one experiment, *n* = 1, PDX-1432C two independent experiments, *n* = 1, 2, PDX-4295 one experiment, *n* = 3, CRCM434 three independent experiments, *n* = 3, 2, 2, CRCM412 four independent experiments, *n* = 4, 2, 4, 4. (**E**) Lung cancer cell biomass captured with cfDNA3 samples, deep needle samples (Needle a) or shallow needle samples (Needle b). One-way Anova followed by Tukey multiple comparisons test, *P* values: CRCM412 cfDNA1/Needle-b = 0.0486, Needle-a/Needle-b = 0.0368. Error bars represent standard deviation (SD) of the mean. Each dot corresponds to a biopsy sample. MDA-MB-231 three experiment, *n* = 1, 4, 4, MDA-MB-468 one experiment, *n* = 5, PDX-4295 two experiments, *n* = 1, 2, CRCM412 three independent experiments, *n* = 2, 1, 4. (**F**) Tumor volume of tumors PDX-1432C at the times of euthanasia and terminal end bleed: T1, T2 and T3 (left). Each dot corresponds to a tumor. Barcode detection in plasma at the different time points, success (green box), unsuccessful (gray box) (right panel). (**G**) Representation of the clonal composition in tumors from clone-splitting experiment (left panels) and their correlation between primary tumor and cfDNA (right panels). PDX-1432C two independent experiments, *n* = 12, 12. For panels (**B–E**) and (**G**), only samples in which barcodes were detected in the plasma were included.

© The Author(s)

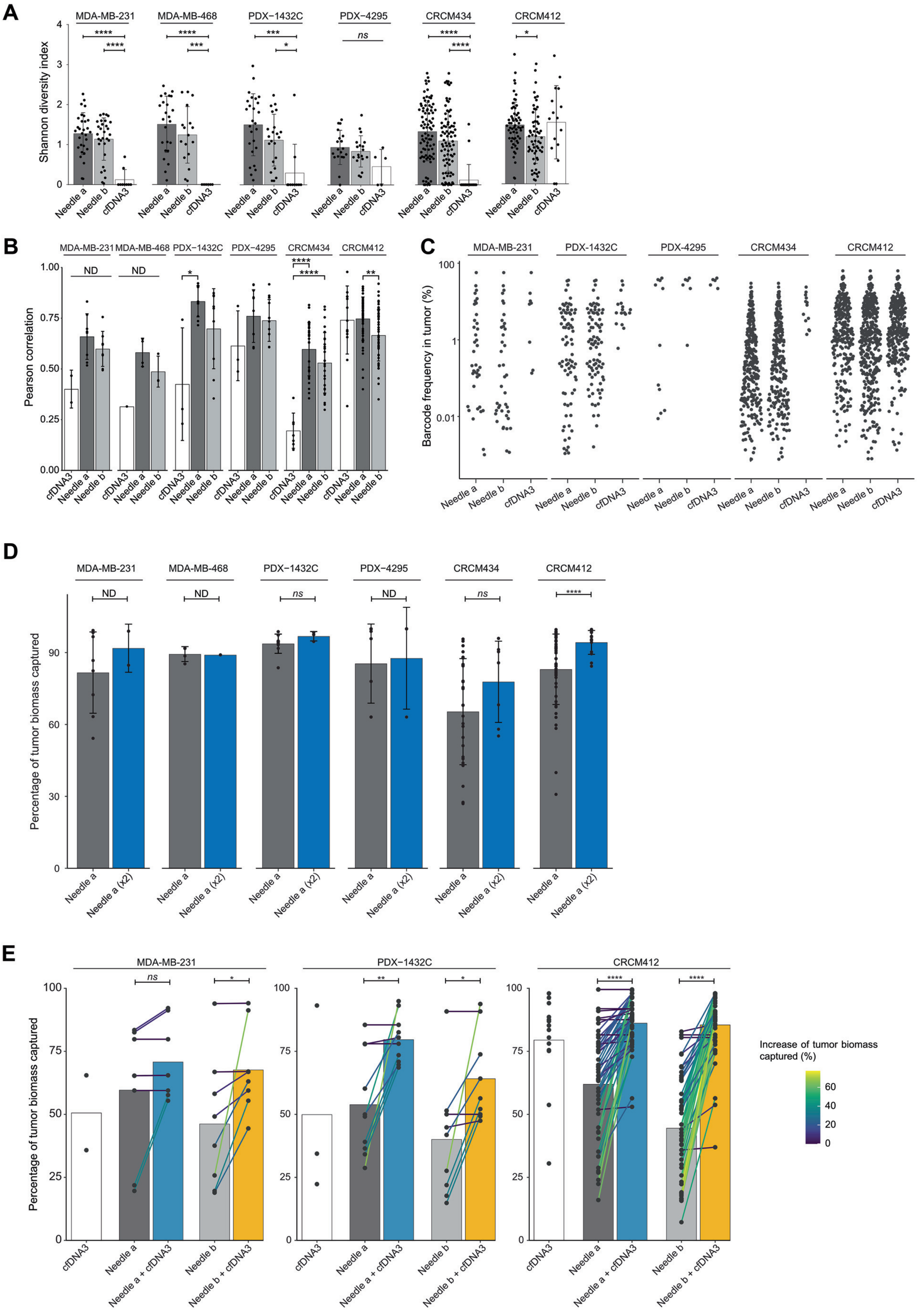
A
MDA-MB-231
MDA-MB-468
PDX−1432C
PDX−4295
CRCM434
CRCM412
Shannon diversity index
Needle a
Needle b
cfDNA3
ns
B
Pearson correlation
ND
C
Barcode frequency in tumor (%)
D
Percentage of tumor biomass captured
Needle a (x2)
E
Needle a + cfDNA3
Needle b + cfDNA3
Increase of tumor biomass captured (%)

 © The Author(s)

Figure EV6. Combination of solid and liquid biopsies.

(**A**) Shannon diversity index from Needle and cfDNA samples. In this panel, all the cfDNA samples were included, including those with unsuccessful barcode recovery. Needle a corresponds to solid biopsy reaching the center of the tumor (deep needle sampling). Needle b corresponds to shallow sampling, covering only a quarter of the tumor diameter. Each dot corresponds to a biopsy sample. One-way ANOVA followed by Tukey multiple comparisons test, *P* values MDA-MB-231 Needle-a/cfDNA3 = 2.4e-07, Needle-b/cfDNA3 = 4.0e-06; MDA-MB-468 Needle-a/cfDNA3 = 3.1e-05, Needle-b/cfDNA3 = 0.0008; PDX-1432C Needle-a/cfDNA3 = 0.0001, Needle-b/cfDNA3 = 0.0113; CRCM434 Needle-a/cfDNA3 = 6.5e-11, Needle-b/cfDNA3 = 1.2e-07; CRCM412 Needle-a/Needle-b = 0.0480. Error bars represent standard deviation (SD) of the mean. MDA-MB-231, three independent experiments, *n* = 1,4,4, MDA-MB-468 one experiment, *n* = 6, PDX-1432C two independent experiments, *n* = 6, 4, PDX-4295 two experiment, *n* = 1, 4, CRCM434, four independent experiments, *n* = 7, 6, 4, 5, CRCM412, four independent experiments, *n* = 4, 2, 5, 5. (**B**) Pearson correlation coefficients measuring the barcode similarity between biopsies and primary tumors (including all pieces). One-way ANOVA followed by Tukey multiple comparisons test, *P* values CRCM434 cfDNA3/Needle-a = 7.9e-09, cfDNA3/Needle-b = 7.3e-07; CRCM412 Needle-a/Needle-b = 0.0032. Error bars represent standard deviation (SD) of the mean. (**C**) Representation of the barcode frequency in primary tumors, for barcodes detected in Needle a (deep needle samples), Needle b (shallow needle samples) and liquid biopsy (cfDNA3). Each dot represents a barcode captured in a biopsy sample. (**B, C**) MDA-MB-231 one experiment, *n* = 2, MDA-MB-468 one experiment, *n* = 1, PDX-1432C two independent experiments, *n* = 1, 2, PDX-4295 one experiment, *n* = 3, CRCM434 three independent experiments, *n* = 3, 2, 2, CRCM412 four independent experiments, *n* = 4, 2, 4, 4. (**D**) Primary tumor biomass captured in each needle sample or in two combined needle samples (blue bars). Student's unpaired *t* test, *P* values: PDX-1432C = 0.1069, CRCM434 = 0.3852, CRCM412 = 3.8e-05. ns: non-significant, *****P* value < 0.0001. Error bars represent standard deviation (SD) of the mean. MDA-MB-231, one independent experiments, *n* = 2, MDA-MB-468 one experiment, *n* = 1, PDX-1432C two independent experiments, *n* = 1, 2, PDX-4295 two experiment, *n* = 1, 2, CRCM434, three independent experiments, *n* = 3, 2, 2, CRCM412, four independent experiments, *n* = 4, 2, 4, 4. (**E**) Primary tumor biomass captured with biopsy methods with barcode frequency in biopsy thresholds at > 1%. Combination of deep needle sample and cfDNA (Needle a + cfDNA3) or shallow needle sample and cfDNA (Needle b + cfDNA3). Correlated needle samples are linked with their associated increased value when cfDNA is added, and the color of the line is scaled on the increase in tumor biomass captured as percentage. Each dot corresponds to a biopsy sample. Paired *t* test, *P* values: MDA-MB-231 Needle-a/Needle-a+cfDN3 = 0.0836, Needle-b/Needle-b+cfDN3 = 0.0279; PDX-1432C Needle-a/Needle-a+cfDN3 = 0.0041, Needle-b/Needle-b+cfDN3 = 0.0101; CRCM412 Needle-a/Needle-a+cfDN3 = 2.0e-12 Needle-b/Needle-b+cfDN3 = 6.8e-18. ns: non-significant, **P* value < 0.05, ***P* value < 0.01, ****P* value < 0.001, **** *P* value < 0.0001. Error bars represent standard deviation (SD) of the mean. MDA-MB-231 one experiment, *n* = 2, PDX-1432C two independent experiments, *n* = 1, 2, CRCM412 four independent experiments, *n* = 4, 2, 4, 4. For panels (**B**, **C**) and (**E**), only samples in which barcodes were detected in the plasma were included.

© The Author(s)

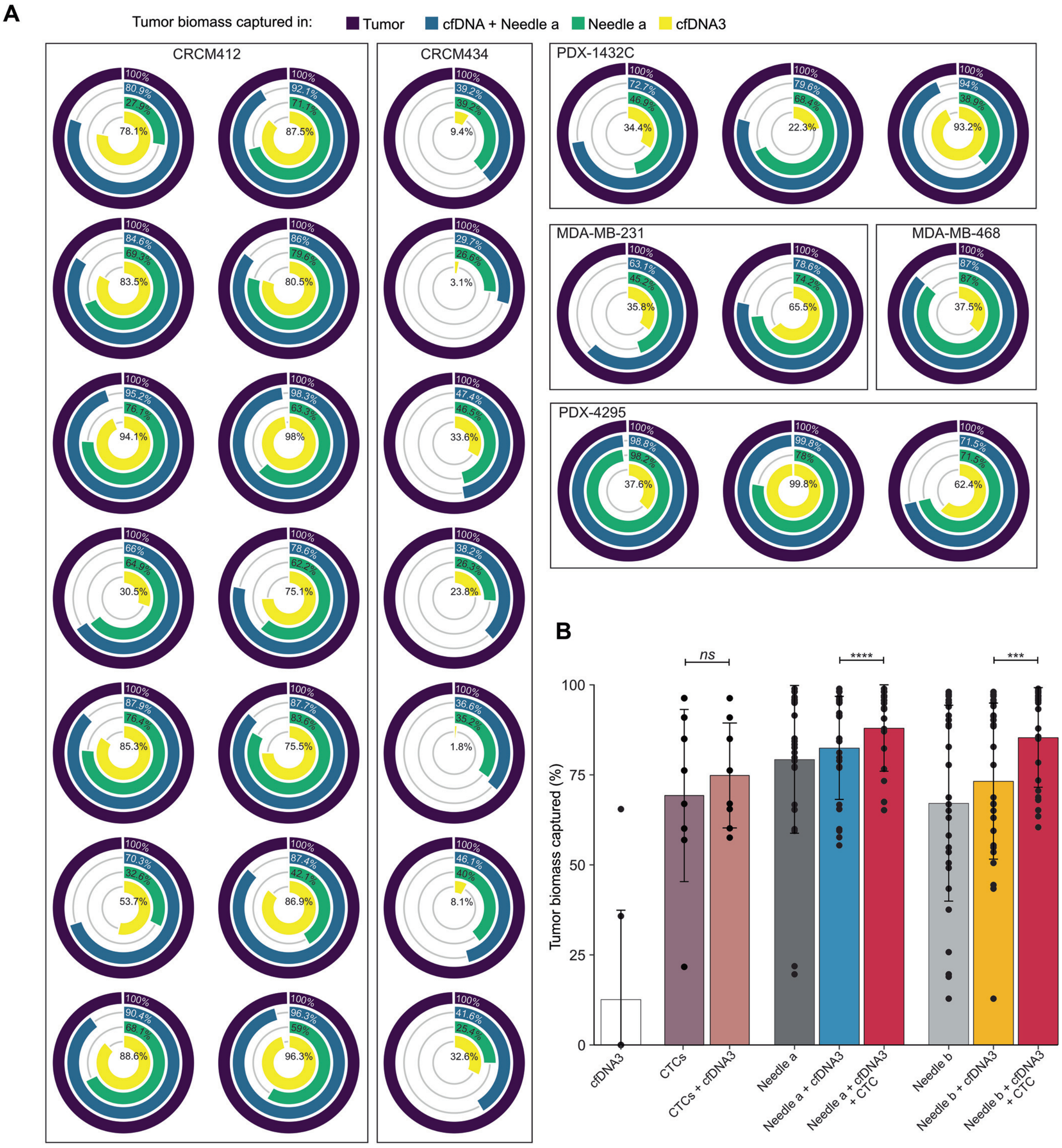

Figure EV7. Analysis of the tumor biomass captured in solid and liquid biopsies.

(A) Circle plots representing the tumor biomass captured by each biopsy sampling alone or in combination, from multiple mice per models. Primary tumor plotted as reference (100%) in the outer circle (purple), inner circles represent the primary tumor biomass captured with cfDNA (yellow), deep needle samples (green) and combination of the two methods (blue). Only barcodes above 1% of the total frequency of biopsy samples were included in the computation of primary tumor biomass. MDA-MB-231 one experiment, $n = 2$, MDA-MB-468 one experiment, $n = 1$, PDX-1432C 2 independent experiments, $n = 1, 2$ PDX-4295 one experiment, $n = 3$, CRCM434 three independent experiments, $n = 3, 2, 2$, CRCM412 four independent experiments, $n = 4, 2, 4, 4$. **(B)** Primary tumor biomass captured with biopsy methods in MDA-MB-231, with barcodes frequency in biopsy thresholds at > 1%. Red bars indicate combinations of needles samples with cfDNA and CTCs. Paired *t* test, *P* values: CTCs/CTCs+cfDNA3 = 0.2530, Needle-a+cfDNA3/Needle-a+cfDN3 + CTCs = 2.1e-06, Needle-b +cfDNA3/Needle-b+cfDN3 + CTCs = 0.0007, ns: non-significant, ****P* value < 0.001, *****P* value < 0.0001. Error bars represent standard deviation (SD) of the mean. MDA-MB-231 two independent experiments, $n = 4, 4$. For these panels, only samples in which barcodes were detected in the plasma were included.

© The Author(s)
